# Resonance assignment of human LARP4A La module

**DOI:** 10.1007/s12104-019-09871-4

**Published:** 2019-01-10

**Authors:** Isabel Cruz-Gallardo, Luigi Martino, Roberta Trotta, Stefano De Tito, Geoff Kelly, R. Andrew Atkinson, Antonio Randazzo, Maria R. Conte

**Affiliations:** 1 Randall Centre for Cell and Molecular Biophysics, King’s College London, London, SE1 1UL UK; 2 Department of Pharmacy, University of Naples Federico II, Naples, Italy; 3 MRC Biomedical NMR Centre, The Francis Crick Institute, London, NW1 1AT UK; 4 Centre for Biomolecular Spectroscopy, King’s College London, London, SE1 1UL UK; 5 Present Address: Department of Chemistry, King’s College London, 7 Trinity Street, London, SE1 1DB UK; 6 Present Address: The Francis Crick Institute, 1 Midland Road, London, NW1 1AT UK; 7 Present Address: Institute of Protein Biochemistry, National Research Council, Via Pietro Castellino 111, 80131 Naples, Italy

**Keywords:** LARP4A, La–module, LARPs, RNA binding protein

## Abstract

Human LARP4A belongs to a superfamily of RNA binding proteins called La-related proteins (LARPs). Whilst being a positive regulator of protein synthesis and a promoter of mRNA stability, LARP4A also controls cell morphology and motility in human breast and prostate cancer cells. All LARPs share a characteristic RNA binding unit named the La–module, which despite a high level of primary structure conservation exhibits a great versatility in RNA target selection. Human LARP4A La–module is the most divergent compared with other LARPs and its RNA recognition properties have only recently started to be revealed. Given the key role of LARP4A protein in cancer cell biology, we have initiated a complete NMR characterisation of its La-module and here we report the assignment of ^1^H, ^15^N and ^13^C resonances resulting from our studies.

## Biological context

Human LARP4A is an RNA binding protein (RBP) involved in mRNA stabilisation and translation enhancement, 3′UTR polyA lengthening and miRNA processing (Maraia et al. 2017; Mattijssen et al. 2017; Nussbacher and Yeo 2018; Yang et al. 2011). As it localises to stress granules, membraneless structures associated with mRNA turnover and protection of mRNA during stress conditions, LARP4A has been suggested to play a role in the stress response (Gilbertson et al. 2018; Yang et al. 2011). LARP4A also regulates cancer cell morphology and motility: its siRNA-mediated depletion has been shown to increase cell migration and invasion, whereas its overexpression promotes cell circularity in breast and prostate cancers (Seetharaman et al. 2016).

LARP4A binds to the 3′polyA tail of mRNAs, and associates to translating ribosomes and protein partners including RACK1 (Receptor for Activated C Kinase) and PolyA binding protein (PABP) (Maraia et al. 2017; Yang et al. 2011). How the cellular functions of LARP4A in RNA and tumour biology are mediated by its molecular associations to RNA targets and/or other proteins remains unclear.

LARP4A possesses a La–module, a unique RNA binding unit conserved across all the members of the La-related proteins (LARPs) superfamily and consisting of two domains, the La motif (LaM) and an RNA recognition motif (RRM1) (Bousquet-Antonelli and Deragon 2009; Maraia et al. 2017). Despite sequence conservation, the RNA targets and functions of the La–modules in different LARPs are quite diverse, but the molecular bases of this versatility remain poorly understood (Maraia et al. 2017). The La–module of the human La protein has been extensively studied at the molecular level and its interactions with the 3′UUU_OH_ tail of the nascent RNA polymerase III transcripts well characterised: the LaM and RRM1 act in synergy to accommodate the 3′UUU_OH_ target, with the LaM establishing the majority of the intermolecular contacts with the RNA (Alfano et al. 2004; Kotik-Kogan et al. 2008; Teplova et al. 2006). An analogous mechanism has been reported for human LARP7 and LARP6 (Maraia et al. 2017; Martino et al. 2015; Uchikawa et al. 2015).

The La–motifs (LaM) of LARPs exhibit a high degree of sequence conservation across the superfamily, particularly in six key residues identified in human La as prime mediators of RNA recognition, namely Q20, Y23, Y24, D33, F35, F55 (human La protein numbering). Intriguingly, in human LARP4A Y24 and F55 are replaced by Cys and Met respectively (Merret et al. 2013). Moreover, primary sequence analysis suggests that LARP4A lacks the otherwise conserved wing 2 loop at the C-terminus of the LaM and contains a short inter domain linker between the LaM and the RRM (Maraia et al. 2017; Martino et al. 2015; unpublished). These distinctive characteristics, divergent from other LARPs, may impact on the RNA binding properties of LARP4A. We therefore set out to unveil the structure and the determinants of RNA recognition of LARP4A, to understand its cellular functions and roles in cancer biology. Here, we report the chemical shift assignments of the backbone and side-chain resonances of LARP4A La–module.

## Methods and experiments

### Protein expression and purification

LARP4A La–module, spanning residues 111–287, was cloned in a pET-Duet1 vector (Novagen) with a hexa-Histidine tag at the N-terminus, followed by a TEV protease cleavage site. The recombinant protein was expressed in *Escherichia* coli Rosetta II cells (Novagen) and uniformly labelled with ^15^N or ^15^N/^13^C in minimal media containing ^15^NH_4_Cl (1 g/L) and ^13^C glucose (2 g/L). The cells were grown to an OD value of 0.6 and induced at 18 °C with 1 mM IPTG (Isopropyl β-d-1-thiogalactopyranoside) overnight. The harvested cells were resuspended in a buffer containing 50 mM Tris pH 8, 300 mM NaCl, 10 mM imidazole, 5% glycerol, one Complete protease inhibitor cocktail tablet (Roche), 2 mM phenylmethylsulfonyl fluoride and lysozyme. After sonication and clarification, the lysate was loaded on a 5 mL His-Trap (GE Healthcare) affinity column and the His-tagged protein was eluted with a gradient from 0 to 300 mM of imidazole. The protein was dialyzed into a buffer comprising 50 mM Tris pH 7.25, 100 mM KCl, 0.2 mM EDTA, 1 mM DTT and digested with TEV protease at 4 °C overnight. To isolate the un-tagged protein from the protease, tags and non-digested protein, the mixture was applied onto a Nickel affinity column (Generon). To eliminate any nucleic acid from the sample, the protein was further purified using a 5 mL Hi-Trap heparin column (GE Healthcare) and eluted with a KCl gradient from 0 to 1 M. The pure protein was dialysed into a buffer containing 20 mM Tris pH 7.25, 100 mM KCl, 0.2 mM EDTA and 1 mM DTT.

## NMR spectroscopy

The ^15^N and ^15^N,^13^C-labeled samples of LARP4A La–module (111–287) were concentrated to 600 µM in 20 mM Tris pH 7.25, 100 mM KCl, 0.2 mM EDTA, 1 mM DTT in 99.8% D_2_O or 10%D_2_O/90%H_2_O as appropriate. All the NMR experiments were performed at 25 °C on Bruker Avance III or NEO NMR spectrometers operating at 700, 800 and 950 MHz, equipped with triple resonance cryoprobes. NMR data were processed with Topspin 3.5pl7 software (Bruker) and NMRPipe/NMRDraw (Delaglio et al. 1995). Assignment was performed with CcpNmr Analysis (Vranken et al. 2005) and/or CARA/NEASY (Bartels et al. 1995) softwares. For the assignment of the backbone resonances a set of experiments including ^1^H-^15^N HSQC, HNCO, HNCA, HN(CO)CA, HNCACB and CBCA(CO)NH was used. The side-chain resonance assignments were determined using ^1^H-^15^N HSQC, ^1^H/^15^N- and ^1^H/^13^C-edited NOESY-HSQC and HCCH-TOCSY spectra (Fesik et al. 1988).

## Extent of assignment and data deposition

The chemical shift assignment for LARP4A La–module has been deposited in the Biological Magnetic Resonance Bank (http://www.bmrb.wisc.edu/), accession number 27666.

**Fig. 1:**
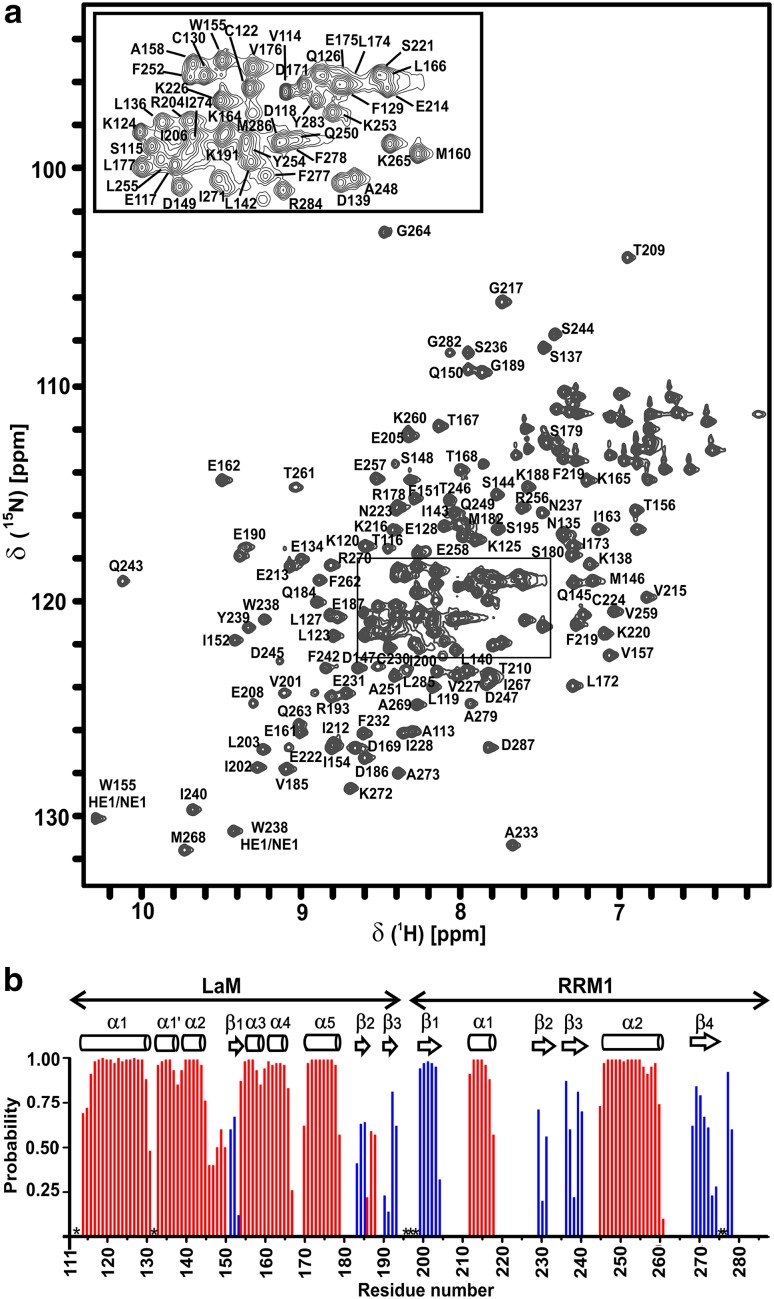
LARP4A La–module NH amide assignment and secondary structure. **a**^1^H-^15^N HSQC spectrum of human LARP4A recorded at 800 MHz and 25 °C. Amide group peaks are labelled with the residue type and numbered corresponding to the protein sequence. A closer view of the central part of the spectrum is shown for clarity. **b** TALOS+ prediction of secondary structure elements for LARP4A La–module. The secondary structure probabilities (red, α-helices; blue, β-strands), plotted against the residue number, are based on backbone HN, N, C′, Cα, and Cβ chemical shifts. Residues for which backbone amide resonance assignments are missing are indicated by asterisks

Human LARP4A La–module displays a well-resolved ^1^H-^15^N HSQC indicating that the protein is folded (Fig. 1a). It comprises 177 residues, with 4 glycine and 8 proline residues. An almost complete backbone assignment (93%) was achieved, identifying 93% of NH (156/168), 87% of Hα (154/177), 97% of Cα (171/177) and 95% of Cβ (165/173) resonances unambiguously. For side chains, 87% of the aliphatic and 48% of the aromatic side-chains (^1^H and ^13^C resonances beyond the Cγ position) were assigned. Resonances of Asn111, Ser132, His234, Asn275, Thr276 and the linker residues (His196–Arg198) could not be assigned. Non-native residues derived from the vector sequence after the His-tag cleavage, a serine and a valine preceding Asn111, were not assigned.

An analysis of the backbone chemical shifts performed with TALOS+ (Shen et al. 2009) revealed that LARP4A La–module contains eight α-helices and seven β-strands distributed between the LaM and RRM1 (Fig. 1b). LARP4A LaM displays the same secondary structure topology found in other LaMs previously described (Alfano et al. 2004; Martino et al. 2015): α1(115–129)–α1′(133–137)–α2(139–145)–β1(151–153)–α3(155–158)–α4(161–165)–α5(170–178)–β2(183–185)–β3(190–193). Likewise, the RRM1 harbours the canonical topology for this class of domains (Clery et al. 2008): β1(200–204)–α1(212–218)–β2(229–233)–β3(236–242)–α2(247–259) β4(267–274).

The backbone and side-chains chemical shifts of the isolated LaM (111–196) and RRM1 (196–287) were also analysed, revealing that they remained largely unchanged in the context of the La–module, and suggesting that the two domains do not adopt a rigid orientation relative to one other in solution.
